# Supplementary material for: Long-term adult human brain slice cultures as a model system to study human CNS circuitry and disease
Source: eLife. 2019 Sep 9;8:e48417. doi: 10.7554/eLife.48417 (PMC6733599; doi:10.7554/eLife.48417)
Supplement: Transparent reporting form [file elife-48417-transrepform.pdf]

eLIFE
1st Floor
24 Hills Road
Cambridge CB2 1JP, UK
P 01223 855340
W elifesciences.org
T @elife

eLife's transparent reporting form

We encourage authors to provide detailed information *within their submission* to facilitate the interpretation and replication of experiments. Authors can upload supporting documentation to indicate the use of appropriate reporting guidelines for health-related research (see EQUATOR Network), life science research (see the BioSharing Information Resource), or the ARRIVE guidelines for reporting work involving animal research. Where applicable, authors should refer to any relevant reporting standards documents in this form.

If you have any questions, please consult our Journal Policies and/or contact us: editorial@elifesciences.org.

Sample-size estimation

- You should state whether an appropriate sample size was computed when the study was being designed
- You should state the statistical method of sample size computation and any required assumptions
- If no explicit power analysis was used, you should describe how you decided what sample (replicate) size (number) to use

Please outline where this information can be found within the submission (e.g., sections or figure legends), or explain why this information doesn't apply to your submission:

The main focus of the presented work was to investigate whether overall morphological integrity, complex cytoarchitecture and electrophysiological properties of human pyramidal neurons are subject to significant changes or whether these features are maintained in long-term human cortical brain slice cultures. Due to the descriptive nature of this study, an explicit power analysis was not applicable. For a systematic and representative analysis we aimed to include tissue from at least 10 patients (finally data from 15 patients were included) and a total of more than 50 neurons (final count: 22 interneurons and 36 pyramidal neurons were electrophysiologically analyzed of which 24 pyramidal neurons were sufficiently filled with biocytin to enable subsequent morphological reconstruction; additionally, 23 GFP labeled pyramidal neurons were included for morphological analysis).

Replicates

- You should report how often each experiment was performed
- You should include a definition of biological versus technical replication
- The data obtained should be provided and sufficient information should be provided to indicate the number of independent biological and/or technical replicates
- If you encountered any outliers, you should describe how these were handled
- Criteria for exclusion/inclusion of data should be clearly stated
- High-throughput sequence data should be uploaded before submission, with a private link for reviewers provided (these are available from both GEO and ArrayExpress)

Please outline where this information can be found within the submission (e.g., sections or figure legends), or explain why this information doesn't apply to your submission:

eLife Sciences Publications, Ltd is a limited liability non-profit non-stock corporation incorporated in the State of Delaware, USA, with company number 5030732, and is registered in the UK with company number FC030576 and branch number BR015634 at the address 1st Floor, 24 Hills Road, Cambridge CB2 1JP | August 2014

All analyzed pyramidal neurons are displayed in Figures 3 and 4.
Each neuron represents a unique entity contributing to a total of 36 (electrophysiological analysis) (Figure 1) and 47 (24 biocytin and 23 GFP labeled pyramidal neurons for morphological analysis) biological replicates (Figures 3, 4, 5 and 7). All biocytin filled pyramidal neurons displaying clear presence of both apical and basal dendritic compartments were included, while incompletely filled neurons were excluded from analysis. These inclusion/exclusion criteria are provided in Results within the "Dendritic morphology of pyramidal neurons *in vitro*" section.

Statistical reporting

- Statistical analysis methods should be described and justified
- Raw data should be presented in figures whenever informative to do so (typically when N per group is less than 10)
- For each experiment, you should identify the statistical tests used, exact values of N, definitions of center, methods of multiple test correction, and dispersion and precision measures (e.g., mean, median, SD, SEM, confidence intervals; and, for the major substantive results, a measure of effect size (e.g., Pearson's r, Cohen's d)
- Report exact p-values wherever possible alongside the summary statistics and 95% confidence intervals. These should be reported for all key questions and not only when the p-value is less than 0.05.

Please outline where this information can be found within the submission (e.g., sections or figure legends), or explain why this information doesn't apply to your submission:

We provide an "Analysis and Statistics" section integrated in Methods: Statistical analyses were performed with Graph Pad Prism 7 using paired or unpaired Mann Whitney test to compare two groups and Kruskal-Wallis test with Dunn's Multiple comparisons test for three groups. Mean values ± standard error of the mean (SEM) or Mean values ± standard deviation (SD) are presented in text and Figures as indicated. Linear regressions were analyzed for the correlations between properties and the days *in vitro* and the distance to the pia (DTP).

(For large datasets, or papers with a very large number of statistical tests, you may upload a single table file with tests, Ns, etc., with reference to sections in the manuscript.)

Group allocation

- Indicate how samples were allocated into experimental groups (in the case of clinical studies, please specify allocation to treatment method); if randomization was used, please also state if restricted randomization was applied
- Indicate if masking was used during group allocation, data collection and/or data analysis

Please outline where this information can be found within the submission (e.g., sections or figure legends), or explain why this information doesn't apply to your submission:

The main focus of the presented work was to investigate whether overall morphological integrity, complex cytoarchitecture and electrophysiological properties of human pyramidal neurons are subject to significant changes or whether these features are maintained in long-term human cortical brain slice cultures. Since each neuron represents a unique entity contributing to a total of 47 (morphological analysis) (Figures 3, 4, 5 and 7) and 36 (electrophysiological analysis) (Figure 1) biological replicates all data points are shown. Group allocation were performed additionally as indicated.

Additional data files ("source data")

- We encourage you to upload relevant additional data files, such as numerical data that are represented as a graph in a figure, or as a summary table
- Where provided, these should be in the most useful format, and they can be uploaded as "Source data" files linked to a main figure or table
- Include model definition files including the full list of parameters used
- Include code used for data analysis (e.g., R, MatLab)
- Avoid stating that data files are "available upon request"

Please indicate the figures or tables for which source data files have been provided:

Source data files have been provided for Figures 3, 4, 5, 6 and 7.
